# MicroRNAs Regulating Mitochondrial Function in Cardiac Diseases

**DOI:** 10.3389/fphar.2021.663322

**Published:** 2021-05-28

**Authors:** Guang-Qiong Zhang, Sheng-Quan Wang, Yan Chen, Ling-Yun Fu, Yi-Ni Xu, Ling Li, Ling Tao, Xiang-Chun Shen

**Affiliations:** ^1^ The State Key Laboratory of Functions and Applications of Medicinal Plants, School of Pharmaceutical Sciences, Guizhou Medical University, Guizhou, China; ^2^ The High Efficacy Application of Natural Medicinal Resources Engineering Center of Guizhou Province, School of Pharmaceutical Sciences, Guizhou Medical University, Guizhou, China; ^3^ The Key Laboratory of Optimal Utilization of Natural Medicine Resources, School of Pharmaceutical Sciences, Guizhou Medical University, Guizhou, China

**Keywords:** microRNAs, mitochondrial function, cardiac disease, cardiac apoptosis, cardiac hypertrophy, diabetic cardiomyopathy, heart failure

## Abstract

Mitochondria are the key organelles that supply cellular energy. As the most active organ in the body, the energy required to maintain the mechanical function of the heart requires a high quantity of high-quality mitochondria in cardiomyocytes. MicroRNAs (miRNAs) are single-stranded noncoding RNAs, approximately 22 nt in length, which play key roles in mediating post-transcriptional gene silencing. Numerous studies have confirmed that miRNAs can participate in the occurrence and development of cardiac diseases by regulating mitochondrial function-related genes and signaling pathways. Therefore, elucidating the crosstalk that occurs between miRNAs and mitochondria is important for the prevention and treatment of cardiac diseases. In this review, we discuss the biogenesis of miRNAs, the miRNA-mediated regulation of major genes involved in the maintenance of mitochondrial function, and the effects of miRNAs on mitochondrial function in cardiac diseases in order to provide a theoretical basis for the clinical prevention and treatment of cardiac disease and the development of new drugs.

## Introduction

Cardiovascular diseases (CVDs) refer to a group of diseases that affect cardiac and vascular tissues and are the most common causes of morbidity and mortality worldwide. By 2030, an estimated 23.6 million people will die due to CVD-related complications annually, and cardiac diseases and stroke are the two leading causes of CVD-associated death (World Health Organization, 2019). Cardiac diseases include multiple disorders, such as pathological cardiac remodeling (Schirone et al., 2017), diabetic cardiomyopathy (Paolillo et al., 2019), heart failure (Rossignol et al., 2019), and myocarditis (Ammirati et al., 2020). Recent studies have indicated that microRNAs (miRNAs) and mitochondria are two important factors involved in the pathophysiological processes underlying cardiac diseases. The targeted regulation of genes by miRNAs is closely associated with mitochondrial function, and miRNAs play important roles in the occurrence and development of cardiac diseases (Borralho et al., 2015; Song et al., 2019). Therefore, a broad understanding of the crosstalk that occurs between miRNAs and mitochondrial function is vital for the prevention and treatment of cardiac diseases.

Mitochondria serve as the cellular “energy factory,” supplying intracellular energy to all cells, which synthesize energy-rich phosphate bonds in the form of adenosine triphosphate (ATP) *via* oxidative phosphorylation (OXPHOS) and are deeply integrated into multiple cellular metabolisms and signaling pathways (Tahrir et al., 2019). As the most metabolically active organ of the body, the heart requires enormous amounts of energy to maintain systolic and diastolic functions, which requires a high content of cardiac mitochondria. Under normal physiological conditions, 90% of the energy required by the heart is derived from mitochondrial OXPHOS, and normal mitochondrial function is a necessary prerequisite for the maintenance of the heart pumping function. Recently, increasing data have shown that mitochondrial dysfunction is involved in the occurrence of cardiac diseases, such as cardiac hypertrophy, myocardial fibrosis (Chen et al., 2019), diabetic cardiomyopathy (Gollmer et al., 2020), and heart failure (Kumar et al., 2019). Accumulating evidence has also suggested that targeting mitochondrial function may represent a promising potential therapeutic strategy for addressing heart failure (Brown et al., 2017). Therefore, reviewing the factors that affect mitochondrial function and cardiac regulatory pathways is important for targeting the clinical treatment of cardiac diseases.

MiRNAs are a class of small, endogenous, single-stranded, noncoding RNAs, and mature miRNAs in eukaryotic cells are approximately 22 nt in length. MiRNAs can inhibit mRNA translation or regulate transcriptional degradation by binding to the 3′untranslated region (UTR) of the target gene, regulating gene expression (Akhtar et al., 2016). The first miRNA, lin-4, was discovered in 1993 (Lee et al., 1993), and miRNA research has continued for more than 20 years. MiRNAs are among the best-studied class of noncoding RNA and are viewed as important regulators of cell differentiation, growth, proliferation, and apoptosis (Colpaert and Calore, 2019), and then participated in a variety of cardiovascular diseases, such as atherosclerosis (Neth et al., 2013), myocardial remodeling (Zhou et al., 2013) and acute myocardial infarction (Zhang et al., 2020). Recent experimental and clinical data have revealed that the differential expression of miRNAs affects mitochondrial function through the regulation of relevant signaling pathways and mitochondrial function-related proteins. Many miRNAs are thought to be involved in the development of CVDs, such as miR-20b, which promotes cardiac hypertrophy by targeting the inhibition of mitofusin 2 (MFN2)-mediated intercellular Ca^2+^ interactions in cardiomyocytes (Qiu et al., 2020). The knockout of miR-181c upregulated the expression of mitochondrial calcium uptake 1 (MICU1), and the regulation of mitochondrial calcium uptake in the myocardium has been shown to protect against heart injury (Banavath et al., 2019). Therefore, understanding the crosstalk between miRNAs and mitochondrial function is important for the study of cardiac diseases and the development of potential drug interventions.

We reviewed the existing miRNA research associated with mitochondrial function and cardiac disease (myocardial apoptosis, cardiac hypertrophy, diabetic cardiomyopathy, and heart failure) found in an English database and attempted to summarize identified miRNAs thought to be involved in the regulation of mitochondrial function. In particular, we focused on the miRNA-mediated regulation of target genes and related signaling pathways involved in mitochondrial function and are thought to affect cardiac homeostasis. We also attempted to identify existing gaps in the current research, including the challenges encountered in miRNA research. We aimed to provide a better understanding of the potential mechanisms that link miRNAs and mitochondria in the development of cardiac diseases and to provide novel targets for the early diagnosis, prevention, and treatment of cardiac diseases.

## MicroRNAs Biogenesis

### Canonical MicroRNAs

With the advent of deep sequencing technology, a large number of noncoding miRNAs have been identified, and our understanding of complex miRNAs has increased. The biogenesis of miRNAs occurs in both the nucleus and cytoplasm through multiple enzymatic cleavage steps (Kim Y.-K. et al., 2016). MiRNAs are encoded by genomic DNA and are transcribed by RNA polymerase II (POL II) or POL III, which produce longer primary miRNA transcripts, known as pri-miRNAs, which can be up to 1,000 nt in length (O’Donnell et al., 2005; Cai et al., 2004). Subsequently, the stem-loop structure of pri-miRNAs is modified by the RNase class III enzyme DROSHA to form precursor-miRNAs (pre-miRNAs) by truncating the stem-loop, while the RNA-binding protein DGCR8 assisted DROSHA in acting on miRNAs to form a hairpin-loop structure pre-miRNA that is approximately 70–100 nt in length. DGCR8 and DROSHA are collectively known as the microprocessor complex, resulting in the RNase III-mediated cleavage of 2-nt-3′ overhangs (Lee et al., 2003; Denli et al., 2004; Lund et al., 2004; Han et al., 2006; Ha and Kim, 2014; O’Brien et al., 2018). These processes all occur in the nucleus.

**FIGURE 1:**
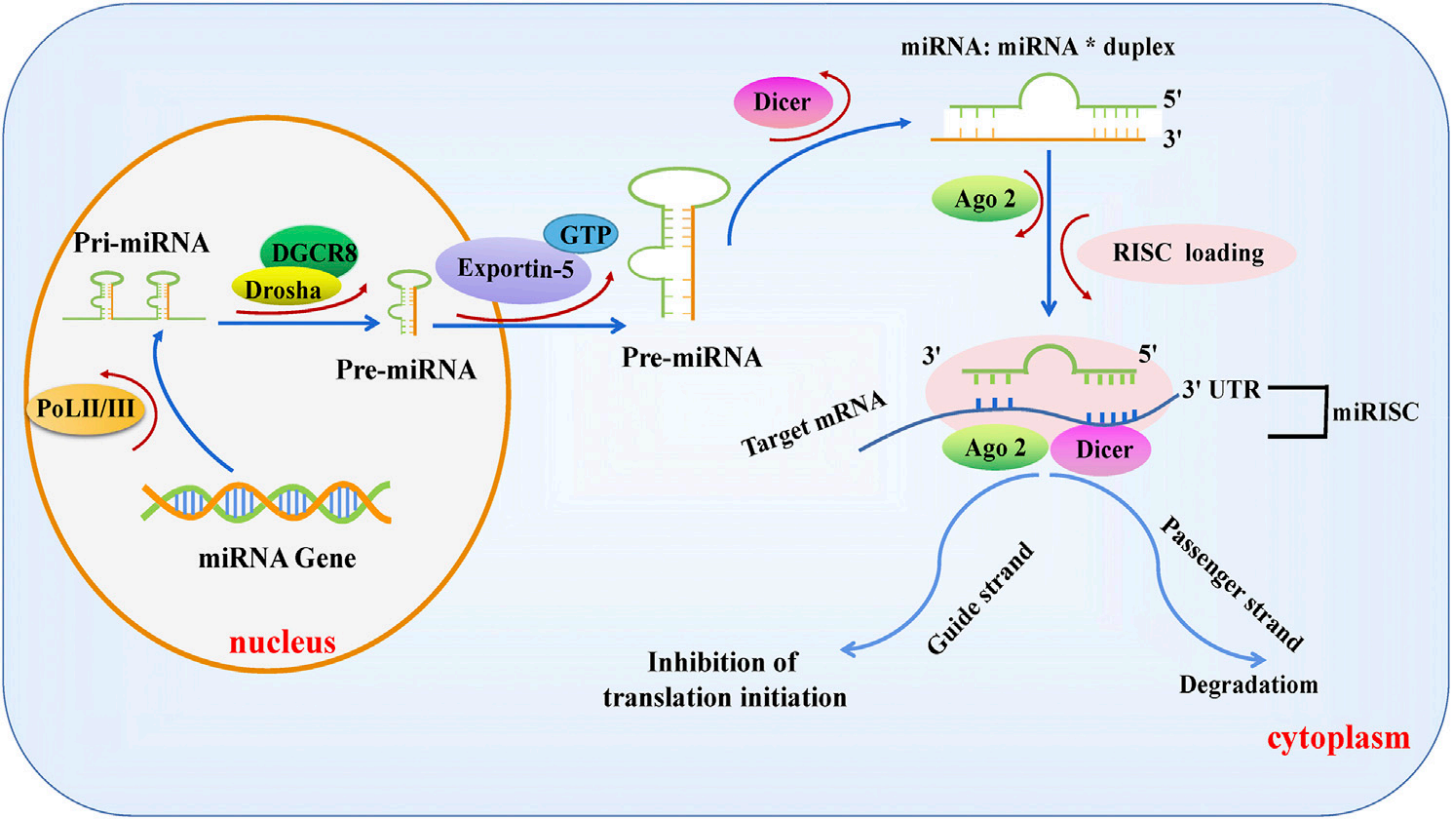
Biogenesis of canonical microRNAs. MiRNA genes are transcribed into a single-stranded primary miRNA by POL II or POL III, followed by the modification of the stem-loop structure of pri-MiRNAs by DROSHA, resulting in the formation of a 70–100 nt pre-miRNA with the assistance of the RNA-binding protein DGCR8. Subsequently, pre-miRNAs are exported to the cytoplasm by Ran GTPase and Exportin-5 and cleaved by Dicer to form mature miRNA double strands, one of which, known as the guide strand, is introduced into the RISC complex containing AGO. The other strand, called the passenger chain, is degraded. The miRNA-loaded RISC combines with the reverse complementary sequence within the 3′UTR of the target mRNA, suppressing the translation or degradation of the target mRNA.

Pre-miRNAs are transported into the cytoplasm by Exportin-5, which depend on Ran gtpase (a GTP-binding nuclear protein) (Yi et al., 2003), and the terminal loops of pre-miRNAs are removed by the enzymatic cleavage of the RNase III Dicer to form a 22-nt miRNA: miRNA* duplex with incomplete complementarity (Hutvágner et al., 2001; Ketting et al., 2001). One of the stranded molecules is introduced into the RNA-induced silencing complex (RISC) containing argonaute proteins (AGOs), known as a guiding strand. At the same time, we see the degradation of the passenger strand. miRNA-loaded RISCs (miRISCs) bind to reverse complementary sequences within the 3’ UTR of the target gene mRNA, inhibiting the translation or degradation of target gene mRNAs and inducing gene silencing. Figure 1 shows the canonical synthesis process for miRNAs (Martinez et al., 2002; Pratt and MacRae, 2009; Winter et al., 2009; Hammond, 2015; Wilson et al., 2015; Bartel, 2018).

### Non-Canonical MicroRNAs

**FIGURE 2:**
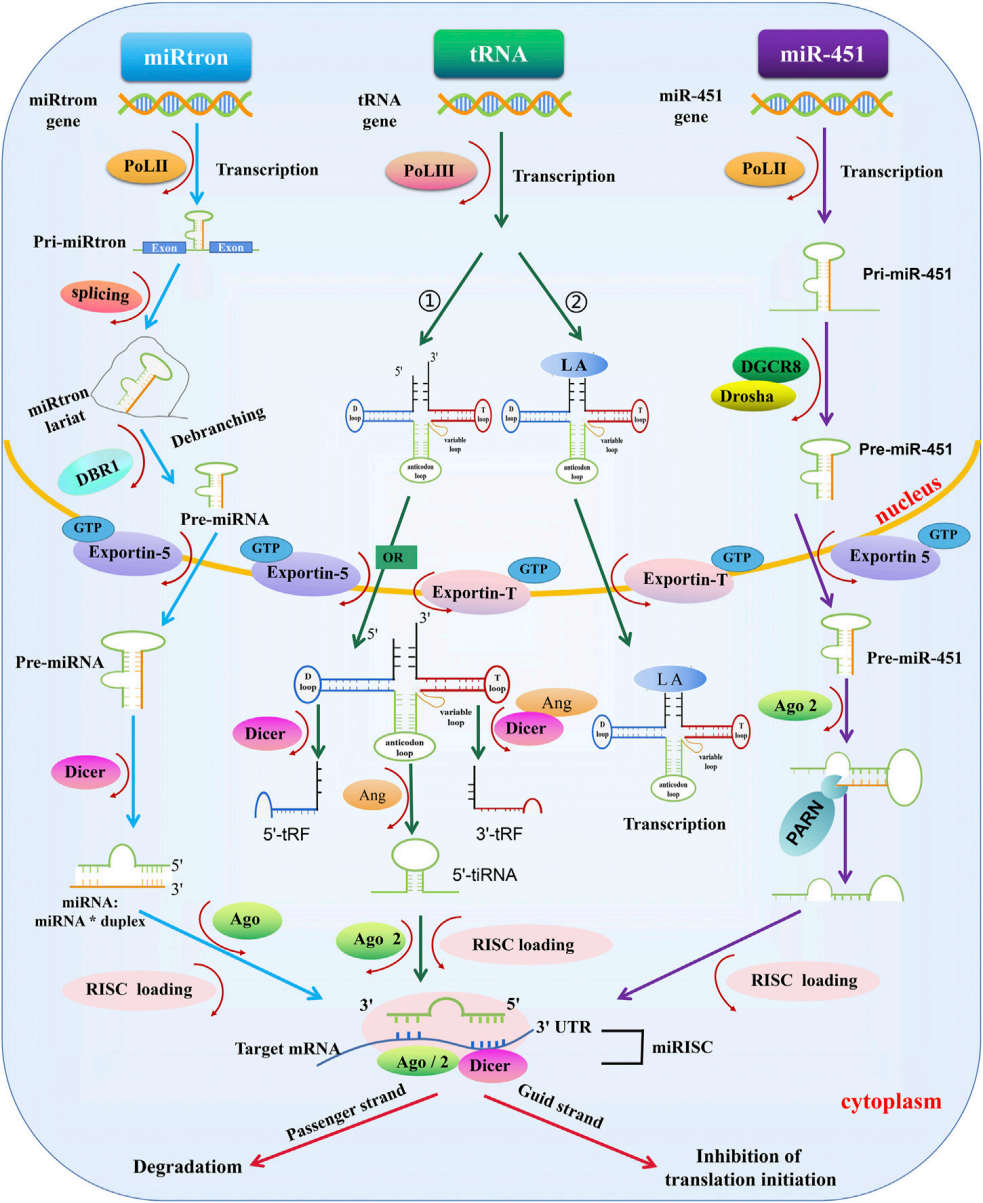
Biogenesis of non-canonical miRNAs. miRtrons: miRtron are primarily localized in the introns of host genes and modified introns, which are formed by the spliceosome and lariat debranching enzyme 1 (DBR1) treatment in the nucleus, can act as pre-miRNAs—which are processed independently of DROSHA/DGCR8. The remaining miRtron biosynthesis process is the same as canonical miRNAs. tRNA: 1) tRNA starts from POL III in the early stage. Next, the pre-tRNA is exported to the cytoplasm by Exportin-5 or Exportin-T. In the cytoplasm, the pre-tRNA adopts a cloverleaf-shaped secondary structure, composed of a D-loop, an anticodon loop, a T-loop, and a variable loop. Pre-tRNA is cleaved by Dicer or angiogenin (ANG): the h D-loop is cleaved by Dicer, the T-loop is cleaved by Dicer and ANG, and the anticodon loop is cleaved by ANG. Some tRNA fragments are loaded into RISC containing AGO2 protein and regulate gene expression. 2) tRNAs transcribed and synthesized by POL III can be stabilized by LA and transported to the cytoplasm under the action of Exportin-T and Ran GTPase to maintain the stability of tRNAs for translation. MiR-451: MiR-451 in transcribed by POL II. After modification by DROSHA/DGCR8 treatment, miR-451 is exported to the cytoplasm by Exportin-5 and Ran GTPase, cleaved by an unknown endonuclease at the 3′end, and loaded into the RISC-AGO complex to exert its biological function, a process independent of Dicer enzymatic cleavage.

In addition to the canonical pathway, some miRNAs are produced through alternate biogenesis pathways and are similar in structure and function to the miRNAs. These are called non-canonical miRNAs: e.g., miRtrons, transfer RNAs (tRNAs), and miR-451 (Martinez et al., 2002; Abdelfattah et al., 2014). Although non-canonical miRNAs may skip one or more steps of the canonical synthesis pathway, Dicer appears to be essential for non-canonical miRNAs, except for miR-451 (Okamura et al., 2007; Ruby et al., 2007; Cheloufi et al., 2010; Abdelfattah et al., 2014; Stavast and Erkeland, 2019) (Figure 2).

#### MiRtrons

MiRtrons were the first identified miRNAs synthesized through non-canonical pathways in *Drosophila melanogaster* and *Caenorhabditis elegans*. MiRtrons are coded by the introns of host genes (Okamura et al., 2007; Wen et al., 2015). Through splice cleavage events, the intron lariat is debranched by lariat debranching enzyme 1 (DBR1), and the modified intron can serve as a pre-miRNA, independent of DROSHA/DGCR8 (Westholm and Lai, 2011). The next step in the synthesis process is consistent with the canonical miRNAs synthesis pathway. (Babiarz et al., 2008).

#### MicroRNAs From Transfer RNAs

The structure of tRNAs differs from that of other miRNAs. A mature tRNA is processed by POL III during the early stage. The pre-tRNA adopts a cloverleaf-shaped secondary structure, composed of a D-loop, an anticodon loop, a T-loop, and a variable loop (Oberbauer and Schaefer, 2018). Next, the pre-tRNA is exported to the cytoplasm by Exportin-5 or Exportin-T, where the pre-tRNA is cleaved by Dicer or angiogenin (ANG): Dicer cleaves the D-loop, both Dicer and ANG cleave the T-loop, and ANG cleaves anticodon loop. The cleaved tRNA interacts with AGO to form a mature miRNA complex involved in a variety of biological functions (Cao et al., 2020). In addition, the lupus autoantigen (LA) is a conserved nuclear RNA-binding factor. The 3′-oligo (U) of POL III is a sequence-specific signal for the termination POL III transcription and a high-affinity binding site for LA, and LA is closely involved in tRNA synthesis associated with POL III transcription. tRNAs that are transcribed and synthesized by POL III can be stabilized by LA and transported to the cytoplasm by Exportin-T and Ran gtpase, which maintain the stability of tRNAs for translation. LA also plays a major role in the inhibition of Dicer-mediated 5′-tRNA-derived RNA fragment cleavage (Okamura et al., 2007; Bayfield and Maraia, 2009; Blewett and Maraia, 2018; Stavast and Erkeland, 2019).

#### MiR-451

Unlike other miRNAs, the biosynthesis pathway of miR-451 is independent of Dicer but requires the AGO2 slicer activity to achieve miRNA maturation (Cheloufi et al., 2010; Cifuentes et al., 2010). MiR-451 originates from the transcription of its host gene by POL II, generating pri-miR-451, which is modified by DROSHA/DGCR8 in the nucleus and transformed into pre-miR-451. Following export to the cytoplasm by Exportin-5 under the action of Ran gtpase (Stavast and Erkeland, 2019), pre-miR-451 cannot be cleaved by Dicer because the stem-loop structure is too short. Instead, pre-miR-451 is cleaved by an unknown endonuclease at the three’ end, loaded into AGO2, and subsequently integrated into a RISC complex to perform its function (Abdelfattah et al., 2014). The specificity of miR-451 has encouraged the exploration of Dicer-independent miRNAs.

Increasing investigations have revealed additional Dicer-independent miRNAs, such as miR-1225 and miR-1228, the biogenesis of which are not only independent of Dicer but also DGCR8, Exportin-5, and AGO2 (Havens et al., 2012). In addition, some miRNAs are synthesized through non-canonical pathways, such as snoRNA-derived miRNAs (sno-miR-28) and miRNAs derived from endogenous short hairpin RNAs (miR-320 and miR-484). A more in-depth description of the biosynthetic pathways associated with non-canonical miRNAs can be found elsewhere (Okamura et al., 2007; Stavast and Erkeland, 2019). The biosynthetic pathways that generate miRNAs are very complex, and the mechanisms of miRNA synthesis and the interactions that occur among components require further study. Additional miRNA synthetic pathways likely exist that have not yet been discovered.

## MicroRNAs Regulate Core Mitochondrial Signals

**FIGURE 3:**
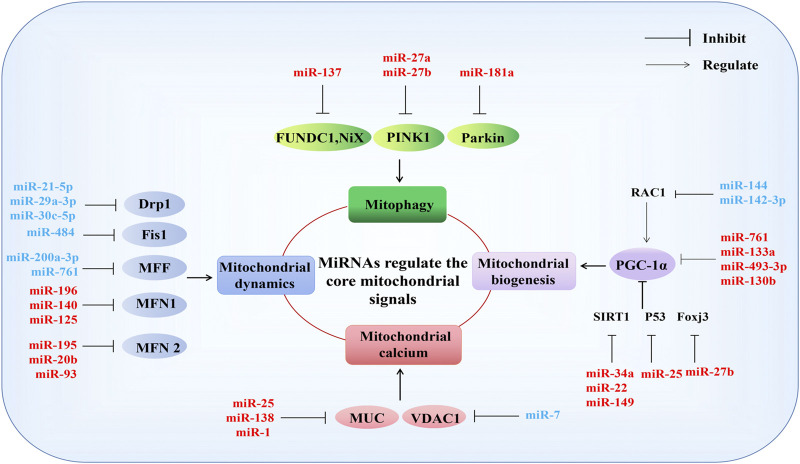
MiRNAs regulate the core mitochondrial signals. MiRNAs participate in mitochondrial function by regulating genes directly related to mitophagy, mitochondrial dynamics, mitochondrial calcium, and mitochondrial biogenesis. Mitochondrial function-related genes: mitophagy (*FUNDC1*, *NiX*, *PINK1*, *Parkin*); mitochondrial dynamics (*Drp1*, *Fis1*, *MFF*, *MFN1*, *MFN2*); mitochondrial calcium (*MUC*, *VDAC1*), mitochondrial biogenesis (*PGC-1α*). See text for detailed explanations. Red and blue represent negative and positive regulation of mitochondrial function, respectively. T represents inhibitory effect, and arrows represent involvement in regulation.

MiRNAs are known to serve as primary regulators of mitochondrial function (Song et al., 2019). Mitochondrial dysfunction can occur due to mitochondrial structural damage, reduced ATP synthesis, the overproduction of reactive oxygen species (ROS), disturbances in calcium ions, mitochondrial DNA (mtDNA) damage, mitochondrial dynamics, biosynthesis (Bose and Beal, 2016), and mitophagy abnormalities (Chistiakov et al., 2018). MiRNAs typically participate in these pathophysiological processes by regulating the expression of mitochondria-related genes (Figure 3).

### Mitochondrial Biogenesis

Mitochondrial biogenesis is a key pathway for intracellular mitochondrial self-renewal and repair. Peroxisome proliferator-activated receptor γ coactivator 1α (PGC-1α) family members and related transcription coactivators are the primary regulators of mitochondrial biogenesis, and PGC-1α can be activated in response to growth signals or energy requirements (Puigserver and Spiegelman, 2003; Zhang and Xu, 2016). Previous evidence has suggested that miR-34a, miR-22, and miR-149 negatively regulate mitochondrial biogenesis by targeting the reduction of Sirtuin 1 (SIRT 1) expression to inhibit the PGC-1α activity (Mohamed et al., 2014; Mao et al., 2019; Xiong et al., 2019). MiR-761, miR-133a, miR-493–3p, and miR-130b negatively regulate mitochondrial biogenesis by directly targeting the inhibition of PGC-1α expression (Xu et al., 2015; Nie et al., 2016; Jiang et al., 2017; Lemecha et al., 2018). MiR-27b and miR-25 inhibit mitochondrial biogenesis by downregulating the expression of forkhead box J3 (Foxj3) and P53, respectively (Wang et al., 2016; Shen et al., 2016). Additionally, miR-144 and miR-142-3p promote mitochondrial biogenesis by targeting the rac family small gtpase 1(Rac1) to activate PGC-1α (Tao et al., 2020; Xia et al., 2020).

### Mitochondrial Dynamics

Mitochondrial dynamics describe how the mitochondria change their shape, distribution, size, and function through the dynamic balance of continuous fission and fusion to meet the energy needs of host cells and respond to environmental changes. In mammals, these dynamic processes are primarily regulated by the dynamin gtpase family. Dynamin-related protein 1 (Drp1), mitochondrial fission factor (MFF), and mitochondrial fission protein 1(Fis1) regulate mitochondrial fission, MFN1 and MFN2 are responsible for mitochondrial outer membrane (OMM) fusion, and optic atrophy 1 (OPA1) mediates mitochondrial inner membrane (IMM) fusion (Tilokani et al., 2018). MiR-21-5p, miR-29a-3p, and miR-30c-5p have been reported to negatively regulate mitochondrial fission Drp1 targeting (Chowdhury et al., 2017; Zhang et al., 2017; Xie et al., 2020). MiR-484 participates in mitochondrial network regulation by targeting Fis1, improving mitochondrial fission (Wang et al., 2012). In addition, miR-200a-3p and miR-761 can improve mitochondrial dysfunction by targeting the negative regulation of MFF, enhancing mitochondrial activity and ATP production (Long et al., 2013; Lee et al., 2017). MFN2-mediated mitochondrial fusion is inhibited by miR-195, miR-20b, and miR-93 (Feng et al., 2019; Purohit et al., 2019; Qiu et al., 2020). Furthermore, miR-196, miR-140, and miR-125 negatively regulate mitochondrial fusion by inhibiting MFN1 (Li J. et al., 2014; Li X. et al., 2014; Ma et al., 2017). However, the direct regulation of OPA1 by miRNAs has not yet been reported and requires further study.

### Mitochondrial Calcium

The ability of mitochondria to act as Ca^2+^ buffers plays a significant role in the regulation of intracellular Ca^2+^ signaling. Mitochondria are the primary store of intracellular calcium, and calcium homeostasis serves as the physiological basis of many cellular activities, such as the regulation of OXPHOS, the modulation of cytosolic Ca^2+^ signals, cell death, secretion, and the production of ROS. The voltage-dependent anion channel (VDAC) in the OMM permits Ca^2+^entry into mitochondria, and the mitochondrial Ca^2+^ uniporter complex (MCU) in the IMM serves as a driving force for the electron transport chain (Jaquenod De Giusti et al., 2018). MiR-25, miR138, and miR-1 have been reported to regulate mitochondrial calcium by targeting MUC, disrupting mitochondrial calcium homeostasis, and miR-7 overexpression improves the release of mitochondrial calcium by targeting VDAC1 (Chaudhuri et al., 2016; Hong Z. et al., 2017; Zaglia et al., 2017).

### Mitophagy

Mitophagy is a specific type of autophagy mode used to clear dysfunctional or redundant mitochondria and serves as an important regulatory mechanism that ensures the quantity and quality of mitochondria, which is necessary for the maintenance of intracellular homeostasis (Vigié and Camougrand, 2017). BCL2-interacting protein three like (BNIP3L, also called Nix), FUN14-domain containing 1 (FUNDC1), PTEN-induced putative kinase 1 (PINK1), and Parkin are key factors that promote the occurrence of mitophagy (Sun T. et al., 2018). MiR-137 negatively regulates mitophagy by targeting FUNDC1 and Nix (Li W. et al., 2014). MiR-27a/b represses mitophagy by downregulating the expression of PINK1 (Kim J. et al., 2016). Additionally, the miR-181a targeting of Parkin has also been found to downregulate mitophagy (Li W. et al., 2014; Cheng et al., 2016; Kim J. et al., 2016).

In summary, the miRNA-mediated regulation of mitochondria-related genes is important for the maintenance of normal mitochondrial function. The heart is the most active organ, and its high demand for energy is met by mitochondrial OXPHOS, which indirectly indicates the importance of miRNA-mediated mitochondrial functional regulation in cardiac disease.

## MicroRNAs Regulate Mitochondrial Function in Cardiac Diseases

Mitochondria account for nearly one-third of the total volume of cardiomyocytes. The homeostasis of mitochondrial function is essential for the maintenance of the normal physiological function of the heart. Abnormal mitochondrial function and quality have been associated with a wide variety of pathological processes that underlie cardiac diseases (Nakamura and Sadoshima, 2018; Picca et al., 2018; Zhou and Tian, 2018). Recent studies have demonstrated that miRNAs regulate mitochondrial functions and participate in cardiac diseases through the direct or indirect targeting of mitochondria-related genes. Table 1 highlights the regulation of mitochondria-related genes by miRNAs in several models of cardiac disease.

**TABLE 1:** MiRNAs involved in cardiac disease. Overview of the mitochondrial functions regulated by miRNAs in several different cardiac phenotypes (myocardial apoptosis, cardiac hypertrophy, diabetic cardiomyopathy, and heart failure).

miRNA	Targets	Model conditions	References
Myocardial apoptosis			
miR-23a	PGC-1α	Dox	Du et al. (2019)
miR-15b-5p	Bmpr1		Wan et al. (2019)
miR-140	MFN 1		Li J. et al. (2014)
miR-29b	Bax		Jing et al. (2018)
miR-140	YES1	HR/IR	Yang et al. (2019)
miR-138	HIF1-α		Liu et al. (2019)
miR-181c	MICU1		Banavath et al. (2019)
miR-30a	P53		Forini et al. (2014); Zhang C. et al. (2019)
miR-486	P53		Sun et al. (2017)
miR-361	PHB1		Wang et al. (2015a)
miR-539	PHB2		Wang et al. (2014)
miR-421	PINK1		Wang et al. (2015c)
miR-145	BNIP3	IR and AR	Li et al. (2012)
miR-324-5p	MTFR1		Wang et al. (2015b)
miR-484	Fis 1		Wang et al. (2012)
miR-761	MFF		Long et al. (2013)
miR-499	CnAα/CnBβ		Wang et al. (2011)
miR-410	HMGB1	LAD and/or HR	Yang et al. (2018)
miR-762	ND2		Yan et al. (2019)
miR-143	C-epsilon		Hong H. et al. (2017)
miR-203	PTP1		Zhang J. et al. (2019)
miR-24	CHOP		Wang and Qian, (2014)
miR-145	PDCD4		Xu et al. (2017)
miR-558	ULK1	ISO	Guo et al. (2019)
miR-208	SOCS2	Sepsis	Ouyang et al. (2020)
Cardiac hypertrophy			
miR-106	MFN 2	Ang Ⅱ/ TAC	Guan et al. (2016)
miR-195-5p			Wang et al. (2019)
miR-20			Sun et al. (2019)
miR-20b			Qiu et al. (2020)
miR-153-3p	MFN 1	ISO/ PAH	Wang et al. (2020)
miR-140			Joshi et al. (2016)
miR-29a-3p	Drp 1	Neuropeptide Y	Xie et al. (2020)
miR-376b-3p	MFF	NA	Sun T. et al. (2018)
miR-485–5p	MAPL	PE	Zhao et al. (2017)
miR-146a	DLST	Ang Ⅱ/TAC	Heggermont et al. (2017)
miR-21	Mt-cytb	SHR	Li H. et al. (2016)
miR-1	MUC	TAC/Chronic exercise	Zaglia et al. (2017)
miR-142–3p	HS2B1	Ang Ⅱ/ AB	Liu et al. (2018)
miR-28	VDAC-1	PE	Kar and Bandyopadhyay, (2018)
Diabetic cardiomyopathy			
Let-7b-5	Mt-cytb	STZ	Li et al. (2019)
miR-92a-2-5	Mt-cytb		Li et al. (2019)
miR-1	Junctin		Yildirim et al. (2013)
miR-141	Slc25a3		Baseler et al. (2012)
miR-30c	PGC-1β	Palmitate and db/db mice	Yin et al. (2019)
miR-1	Fxra	HG	Cheng et al. (2018)
miR-144	Rac-1	HG and STZ	Tao et al. (2020)
Heart failure			
miR-195	SIRT 3	TAC/MI	Zhang et al. (2018)
miR-1a-3p	ND1, COXⅠ	ISO	He et al. (2019)
miR-29	PGC-1α	HF patients	Caravia et al. (2018)
miR-665	GLP1R	LCA	Lin et al. (2019)
miR-152	FLRX5	TAC	LaRocca et al. (2020)
miR-181c	COXⅠ	Plasmid miR-181c *in vivo*	Das et al. (2014)
miR-696	—	TAC	Wang et al. (2017)
miR-532			
MiR-690			
miR-345–3p			

### Cardiomyocyte Apoptosis

Apoptosis refers to programmed death that is regulated by various genes and serves to maintain intracellular homeostasis. Abnormal apoptosis in cardiomyocytes has previously been linked to mitochondrial dysfunction caused by the dysregulation of miRNAs (Makhdoumi et al., 2016). Here, we describe the specific regulation of mitochondrial function by miRNAs in several representative models of myocardial apoptosis.

The cardiomyocyte apoptosis model induced by doxorubicin (Dox) is an antitumor antibiotic that can inhibit many types of tumors; however, its clinical applications are limited due to significant heart toxicity (Shi et al., 2018). In both *in vivo* and *in vitro* Dox-induced cardiomyocyte apoptosis models, miR-23a mimicking was shown to promote mitochondrial fission, cause mitochondrial membrane potential loss, and exacerbate cardiomyocyte apoptosis by targeting the downregulation of PGC-1α and the activation of Drp1 (Du et al., 2019). The overexpression of miR-15b-5p can silence bone morphogenetic protein receptor 1 (Bmpr1) signaling, causing mitochondrial ROS (mtROS) aggregation and resulting in mitochondrial damage, accelerating apoptosis (Wan et al., 2019). MiR-140 inhibits MFN1, causing mitochondrial fission abnormalities and promoting Dox-induced cardiomyocyte apoptosis (Li J. et al., 2014). However, high expression levels of miR-29b can inhibit Bax, reverse the loss of mitochondrial membrane potential, prevent the opening of the mitochondrial permeability transition pore (MPTP) and cardiomyocyte apoptosis (Jing et al., 2018). These data suggest that the regulation of mitochondrial function by miRNAs can have opposing effects, and the in-depth study of their mechanisms of action might result in a therapeutic response to the cardiotoxicity associated with Dox.

Myocardial hypoxia and ischemia/reperfusion injury (HR/IR) models: HR and IR injury models can induce cardiomyocyte apoptosis and myocardial infarction, and miRNA-mediated mitochondrial regulation is involved in these processes. The upregulation of miR-140 and miR-138 improve mitochondrial morphology and reverse IR-induced myocardial apoptosis and myocardial infarction in mice by inhibiting the expression of YES1 and hypoxia-inducible factor 1α (HIF1α) and downregulating the mitochondrial fission proteins Drp1 and Fis1, respectively (Liu et al., 2019; Yang et al., 2019). In addition, the nuclear-encoded miR-181c can be transported to the mitochondria to form an AGO/mt-COX1 complex, which regulates mtROS. The activation of the transcription factor Sp1 can directly upregulate the transcription of MICU1 by binding to the MICU1 promoter region, clarifying the regulatory role of the miR-181c→mt-COX 1→ROS→Sp1→MICU1 signaling pathway in the regulation of mitochondrial calcium uptake in the model; however, the article did not identify the specific target of miR-181c (Banavath et al., 2019). Similarly, in both *in vivo* and *in vitro* models induced by anoxia reoxygenation (AR), HR, or IR, miR-30a, and miR-486 can target p53 transcription, restore the mitochondrial membrane potential and ATP production, improve mitochondrial damage, and inhibit myocardial apoptosis (Forini et al., 2014; Sun et al., 2017; Zhang C. et al., 2019). MiR-361,miR-539, and miR-421 inhibit prohibitin 1 (PHB1), PHB2, and PINK1, respectively, to intensify mitochondrial fragmentation and promote H_2_O_2_-induced cardiomyocyte apoptosis and myocardial infarction (Wang et al., 2014; Wang et al., 2015a; Wang et al., 2015c).

In both *in vitro* and *in vivo* IR and AR-induced models, miR-145 can improve the mitochondrial structure, reduce the production of mtROS, and reverse myocardial apoptosis by targeting BNIP3 (Li et al., 2012). The upregulation of miR-324-5p, miR-484, and miR-761 can improve mitochondrial over fission and inhibit myocardial apoptosis by inhibiting mitochondrial fission regulator 1 (MTFR1), Fis1, and MFF, respectively (Wang et al., 2012; Long et al., 2013; Wang et al., 2015b). The downregulation of miR-499 can inhibit the α and β isoforms of the calcineurin catalytic subunit (CnAα and CnAβ) to activate Drp1, accelerate mitochondrion fission, and promote the occurrence of apoptosis (Wang et al., 2011).

In the left anterior descending artery (LAD)-ligation or HR-mediated myocardial apoptosis models, miR-410 can cause abnormal mitophagy and promote apoptosis by inhibiting high-mobility group box one protein (HMGB1) (Yang et al., 2018). MiR-762 and miR-143 target NADH dehydrogenase 2 (ND2) and protein kinase C-epsilon (PKCε), respectively, resulting in the depletion of the mitochondrial membrane potential and decreased ATP activity, which disrupts mitochondrial function and accelerates myocardial apoptosis (Hong H. et al., 2017; Yan et al., 2019). However, miR-203 can inhibit protein tyrosine phosphatase 1 (PTP1) expression, promote ATP synthesis, restore the mitochondrial membrane potential, and improve myocardial apoptosis (Zhang J. et al., 2019). MiR-24 can inhibit the expression of C/EBP-homologous protein (CHOP) and improve myocardial apoptosis (Wang and Qian, 2014). The overexpression of miR-145 inhibited programmed cell death 4 (PDCD4) expression, improved mitochondrial function, and inhibited myocardial apoptosis in the above experimental models (Xu et al., 2017). In addition, in the apoptotic model induced by isoprenaline (ISO) treatment, miR-558 was able to promote apoptosis by targeting unc-51-like autophagy activating kinase 1 (ULK1), promoting the loss of mitochondrial membrane potential and mtROS overproduction (Guo et al., 2019). In a sepsis-induced mouse model of myocardial apoptosis, the downregulation of miR-208 improved mitochondrial swelling and cell apoptosis by targeting activation of the suppressor of cytokine signaling 2 (SOCS2) (Ouyang et al., 2020).

These data show that the regulation of mitochondrial function by miRNAs has been extensively studied in myocardial apoptosis, especially in models of IR-mediated myocardial apoptosis, which laid a solid foundation for the possibility of using miRNAs as biomarkers in myocardial IR injury-induced cardiomyopathy, although the functions and mechanisms of miRNAs in other induced myocardial apoptosis models require further study.

### Cardiac Hypertrophy

Cardiac hypertrophy is a compensatory response that allows for the maintenance of cardiac function and efficiency under conditions of cardiac overload, which is manifested as the enlargement of the heart and the individual component cardiomyocytes. Physiological hypertrophy often occurs during pregnancy and in athletes, associated with enhanced cardiac contractile function, with no interstitial or alternative fibrosis or cell death, and is usually reversible and does not progress to heart failure. Pathological cardiac hypertrophy is mediated by persistent neuroendocrine hormones and stimulated by mechanical forces and is accompanied by cell death, fibrous remodeling, and mitochondrial dysfunction, which ultimately leads to heart failure (Nakamura and Sadoshima, 2018). Cumulative evidence has demonstrated that miRNA-targeted mitochondrial function and morphological regulation play indispensable roles in cardiac hypertrophy (Jusic et al., 2020).

#### Mitochondrial Fusion-Related MicroRNAs in Cardiac Hypertrophy

MFN1/2 and OPA1 are the main contributors to the process of mitochondrial fusion and are important for maintaining the balance of mitochondrial dynamics. MiR-106 can downregulate the expression of MFN2, exacerbating cardiac hypertrophy induced by transverse aortic constriction (TAC) or angiotensin II (Ang II) pressure overload, which is associated with mitochondrial dysfunction, including mitochondrial cristae defects, the considerable depolarization of the mitochondrial membrane, and mtROS production, suggesting that high miR-106 expression levels promote mitochondrial dysfunction in cardiac hypertrophy (Guan et al., 2016). The same regulatory pathway was also associated with miR-195-5p and miR-20. MiR-195-5p was upregulated *in vitro* and *in vivo* Ang Ⅱ-induced hypertrophic models, and the high expression of miR-195-5p inhibited MFN2 activity, leading to abnormal mitochondrial fusion. MiR-20 is upregulated in hypertrophic cells, causing mitochondrial dysfunction by repressing MFN2 expression, and a similar expression phenomenon has been detected in clinical hypertrophic cardiomyopathy patients (Sun et al., 2019; Wang et al., 2019). Recently, miR-20b was found to be upregulated in an Ang II-induced primary cardiomyocyte hypertrophy model and a TAC-induced hypertrophy model in KM mice, resulting in mitochondrial fragmentation and a sharp decrease in fusion ability. MFN2 plays an important role connecting the sarcoplasmic reticulum to mitochondrial calcium transport, which weakens the mitochondrial buffering capacity and leads to increased cytosolic Ca^2+^, decreased mitochondrial Ca^2+^, and disturbed calcium homeostasis. This phenomenon can be reversed by inhibiting the miR-20b-targeted upregulation of MFN2, showing that the hypertrophic effects of miR-20b can be achieved by the targeted downregulation of MFN2 (Qiu et al., 2020). Additionally, studies have demonstrated that both miR-153-3p and miR-140 are involved in ISO-induced cardiac hypertrophy and ventricular hypertrophy in patients with pulmonary arterial hypertension (PAH), downregulating the expression of MFN1 mRNA and leading to excessive mitochondrial fission (Joshi et al., 2016; Wang et al., 2020). However, the role played by miRNAs in the regulation of OPA1 in cardiac hypertrophy has not yet been reported and represents an unexplored field for researchers looking to study the regulatory effects of miRNAs on mitochondrial fusion.

#### Mitochondrial Fission-Related MicroRNAs in Cardiac Hypertrophy

Drp1/Dynamin I (Dnm1), MFF, and Fis1 are the key genes involved in the regulation of mitochondrial fission. *In vitro*, miR-29a-3p was shown to improve cardiac hypertrophy induced by neuropeptide Y by inhibiting the expression of Drp1 and reducing the expression of the hypertrophy marker gene atrial natriuretic peptide (Xie et al., 2020). In the norepinephrine (NA)-induced cardiac hypertrophy model, the overexpression of miR-376b-3p and the knockdown of MFF attenuated NA-induced mitochondrial fission and cardiac hypertrophy, which suggested that miR-376b-3p could improve mitochondrial fission and reverse cardiac hypertrophy by inhibiting the expression of MFF by binding to its 3’UTR. These results indicated that miR-376b-3p is a novel regulator that affects mitochondrial morphology (Sun Y. L et al., 2018). MiR-485-5p can improve mitochondrial fission and reverse cardiac hypertrophy by activating MFN2 and causes the downregulation of mitochondrial anchored protein ligase (MAPL) in phenylephrine (PE)-induced cardiac hypertrophy models (Zhao et al., 2017). However, whether miRNAs are involved in the growth underlying cardiac hypertrophy by targeting other mitochondrial mitotic proteins, such as Dnm1 and Fis1, requires further exploration.

In hypertrophic cardiac diseases, miRNAs can regulate mitochondrial function through other signaling pathways. For example, the overexpression of miR-146a and the knockout of dihydrolipoyl succinyltransferase (DLST) can both lead to the downregulation of tricarboxylic acid cycle efficiency, causing impaired energy production, similar to the mechanism identified in Ang II- and TAC-induced cardiac hypertrophy and in clinical patients, suggesting that miR-146a and its target DLST are important regulators of the metabolic pathways involved in cardiac hypertrophy (Heggermont et al., 2017). In a spontaneous hypertensive rat (SHR) model, the high expression of miR-21 can rescue mitochondrial dysfunction and cardiac hypertrophy induced by the gene silencing of mtDNA-encoded cytochrome b (mt-Cytb) (Li H. et al., 2016). MiR-1 has been identified as a muscle-specific miRNA, and MCU is a prerequisite for mitochondrial functional homeostasis, ATP synthesis, and cell metabolism. Recently, miR-1 was shown to effectively bind to the 3′UTR of MCU, affecting mitochondrial Ca^2+^ uptake and leading to cardiac hypertrophy. This finding suggests a new role for miR-1/MCU signaling in mitochondrial calcium regulation and cardiac hypertrophy (Zaglia et al., 2017). MiR-142-3p overexpression was shown to silence the energy metabolism regulatory factor Src homology 2 (HS2B1), and protect mitochondrial function by restoring oxygen consumption rate and membrane potential, thus reversing Ang Ⅱ- and abdominal aorta ligation (AB)- mediated cardiac hypertrophy (Liu et al., 2018). MiR-28 can target the mitochondrial gene VDAC1, activate PPARγ, improve mitochondrial function, and induce cardiac hypertrophy mediated by the α1-adrenergic agonist PE *in vitro* (Kar and Bandyopadhyay, 2018).

Although increasing evidence has suggested that miRNAs participate in the development of cardiac hypertrophy by regulating the expression of mitochondria-related genes, more attention has been focused on the pathways involved in mitochondrial fusion and fission. However, the contributions of mitophagy regulation by miRNAs in cardiac hypertrophy should not be ignored and should be further explored.

### Diabetic Cardiomyopathy

Diabetic cardiomyopathy (DCM) is a specific cardiomyopathy, independent of hypertension, coronary heart disease, valvular heart disease, and other cardiac diseases, that occurs in diabetic patients and is associated with ventricular pathological remodeling. DCM is the primary mediator of the increased incidence of heart failure and mortality in diabetic patients (Yin et al., 2019). The regulation of mitochondrial function by miRNAs is actively involved in the development and progression of DCM (Hathaway et al., 2018). In db/db mice and palmitate-induced DCM, the downregulation of miR-30c can inhibit the mitochondrial membrane potential of PPARα by activating PGC-1 β, inducing mitochondrial biogenesis and intensifying the occurrence of DCM (Yin et al., 2019). In the past, let-7 downregulation was shown to improve STZ-induced DCM by regulating the Akt/mTOR signal, but its key target has not been studied in-depth (Li J. et al., 2016).

Recently, both let-7b-5 and miR-92a-2-5p were found to inhibit mtROS overproduction, activate the mitochondrial respiratory chain, prevent lipid deposition, and ameliorate DCM by regulating mt-cytb (Li et al., 2019). In an STZ-induced DCM model, the expression of miR-1 was downregulated, and Junctin expression was upregulated, which induced mitochondrial swelling and the loss of the mitochondrial cistern and granule matrix. Junctin is a downstream target of miR-1, but the specific mechanism of Junctin regulation in DCM remains unclear (Yildirim et al., 2013). However, in a high-glucose (HG)-induced DCM model, the overexpression of miR-1 inhibited Fxra, resulting in the loss of the mitochondrial membrane potential and aggravating DCM damage (Cheng et al., 2018). Therefore, the elucidation of the specific role played by miR-1 and its mechanism of action in DCM is important for the prevention and treatment of DCM. MiR-141 was upregulated in the hearts of STZ-induced FVB mice, which inhibited the translation of mitochondrion phosphate carrier (Slc25a3) without affecting the transcription level, resulting in decreased ATP synthetase activity (Baseler et al., 2012). Recently, the overexpression of miR-144 was found to specifically inhibit Rac-1, regulating the AMPK-PGC-1 α signal, enhancing mitochondrial biogenesis, upregulating the copy number of mtDNA, and restoring the mitochondrial membrane potential, which improved the DCM induced by HG and STZ both *in vitro* and *in vivo* (Tao et al., 2020).

MiRNAs are involved in DCM by affecting mitochondrial function through a wide range of signaling pathways. However, little research has been performed to examine the direct targeting of mitochondrial functional proteins, especially those involved in mitochondrial dynamics and mitophagy.

### Heart Failure

The pathological factors that contribute to heart failure are very complex and are often the cumulative effect of many diseases, including coronary heart disease, hypertension, and ischemic heart disease. Mitochondrial function is an important target for the treatment of heart failure, and the regulation of mitochondrial function by miRNAs contributes to the occurrence and development of heart failure (Pinti et al., 2017). MiR-696, miR-532, miR-690, and miR-345-3p are highly expressed in the mitochondria of C57BL/6 mice with TAC-induced heart failure. Enrichment analysis of these four miRNAs pathways by TarBase showed that these pathways were closely related to mitochondrial genesis, fission, and energy metabolism, which further revealed that the effects of miRNAs on mitochondrial function were closely related to heart failure (Wang et al., 2017). The mitochondrial deacetylase Sirtuin 3 (SIRT3) plays a key role in the maintenance of mitochondrial function by regulating mitochondrial acetylation. In patients with heart failure and TAC/MI- and Ang II-induced heart failure models, the high expression of miR-195 can downregulate SIRT3 activity, aggravating the excessive acetylation of pyruvate dehydrogenase complex (PDC) and ATP synthetase in patients—resulting in disordered mitochondrial energy metabolism (Zhang et al., 2018). Treatment with an miR-1a-3p agomir can improve the symptoms of heart failure in ISO-induced HF mice by increasing the expression of mitochondrial NADH dehydrogenase 1 (ND1) and mitochondrial cytochrome c oxidase I (COX Ⅰ) (He et al., 2019). In addition, in miR-29-deficient mice, cardiac dysfunction, abnormal cardiac metabolism, and the activation of PGC-1 α led to mitochondrial biological disorders. The expression of miR-29 can inhibit PGC-1 α, control mitochondrial biogenesis, and improve heart failure (Caravia et al., 2018). In a heart failure model induced by left coronary artery ligation (LCA), the high expression of miR-665 can reduce mitochondrial ATP enzymatic activity and damage cardiac function, whereas the inhibition of miR-655 can upregulate glucagon-like peptide 1 receptor (GLP1R) and cyclic adenosine monophosphate (cAMP) signaling to stabilize heart failure (Lin et al., 2019). In a TAC-induced heart failure model in C57BL/6 mice, the high expression of miR-152 resulted in the destruction of the mitochondrial ultrastructure and induced cardiac metabolic disorder, which was mediated by the targeted inhibition of FLRX5 (a key factor involved in the regulation of mitochondrial homeostasis). By contrast, the silencing of miR-152 could reverse the above pathological process and improve the heart failure caused by stress overload (LaRocca et al., 2020). In addition, nuclear-encoded miR-181c could be shifted toward mitochondrial targeting to inhibit the expression of the mitochondrial gene *COX1*, resulting in abnormal mitochondrial metabolism, the loss of OXPHOS, and heart failure (Das et al., 2014). These data suggested that miRNAs primarily contribute to heart failure by regulating mitochondrial metabolic pathways. Therefore, contributions of miRNAs on other mitochondrial functions associated with heart failure require additional study.

## Challenges and Development

Since the first miRNA was identified in 1993, research on miRNAs has received a great deal of attention, and miRNAs are the best-studied noncoding RNAs, especially relative to cardiovascular disease (Colpaert and Calore, 2019). One of the most characteristic features of the mitochondria is that it has its own genome, and mtDNA can participate in the synthesis, transcription, and replication of mitochondrial proteins (Gustafsson et al., 2016). Consequently, the discovery of mitochondrial genome-encoded miRNAs, which play key roles in cardiomyopathy, continues (Song et al., 2019). However, some challenges remain to be overcome, which provides avenues for future research:

1) The biosynthetic pathway of miRNAs is very complex, involving interactions among multiple enzymes and complex components. The roles played by each component in the miRNA synthesis pathway remain controversial, and how the passenger strand is degraded remains unclear (Jaé and Dimmeler, 2020). In addition to canonical synthetic pathways, non-canonical synthetic pathways are gradually being uncovered. Although these synthesis pathways bypass one or more steps of the canonical pathway, mature miRNAs synthesized by these pathways function similarly to those synthesized by canonical pathways (Ruby et al., 2007). Therefore, exploring and discovering the biosynthetic pathways used to generate additional miRNAs and elucidating the mechanisms of these miRNAs in cardiac disease remains a difficult and urgent problem to be solved.
2) The regulation of mitochondrial function by miRNAs has received increasing interest, and many mitochondrial-encoded miRNAs and nuclear-encoded miRNAs have been shown to translocate to the mitochondria to perform their biological functions (Fan et al., 2019). However, the extraction of uncontaminated mitochondria remains a challenge; therefore, the biosynthetic pathway and mode of action associated with mitochondrial miRNAs remain unclear. In addition, how nuclear-encoded miRNAs translocate to the mitochondria to perform their functions and the mediators of this process have yet to be determined. Thus, whether the mitochondrial genome can encode miRNAs and how the crosstalk between nuclear-encoded miRNAs and the mitochondria affects the development of cardiac disease remains to be further studied.
3) Generally, a single miRNA can target multiple genes, and multiple miRNAs can regulate the same gene, exerting different biological functions (Selbach et al., 2008). Both miR-106a and miR-20b can induce mitochondrial dysfunction and promote cardiac hypertrophy by inhibiting the expression of MFN2 (Guan et al., 2016; Qiu et al., 2020). MiR-140 improves I/R-induced myocardial apoptosis by inhibiting the expression of Drp1 and Fis1 through the targeting of YES1 (Li J. et al., 2014). However, in the Dox-induced cardiomyocyte apoptosis model, miR-140 was found to mediate cardiomyocyte apoptosis by inhibiting the expression of MFN1 (Li J. et al., 2014). Due to the large number of gene pools associated with mitochondrial function and the complex pathogenic factors that underlie cardiac diseases, research has both increased our understanding of the specific mechanisms and targets through which miRNAs regulated mitochondrial activity in cardiac diseases and introduced new challenges—which can be used to guide our future experimental studies.
4) MiRNA is also widely involved in other cardiac diseases, such as coronary artery disease (Hinkel et al., 2014), myocardial fibrosis (Yuan et al., 2017), and congenital heart disease (Smith et al., 2015). However, there is a lack of research on miRNA involved in these diseases by regulating mitochondrial function. Therefore, it is an urgent need to actively explore and study the specific regulatory mechanism of miRNA regulating mitochondrial function in more cardiac diseases.

## Conclusion

Cardiovascular diseases are among the leading causes of death, and mitochondria represent promising potential therapeutic targets for cardiovascular diseases. The emergence of miRNAs has been a major breakthrough in the study of cardiac disease. In recent years, the miRNA-mediated regulation of the mitochondrial network has received much attention and has been verified in cardiac diseases. This review summarizes the biogenesis of miRNAs, their regulatory roles in mitochondrial functions, and the current research regarding miRNAs in cardiac disease, indicating that the regulation of mitochondrial function by miRNAs plays an important role in cardiomyocyte apoptosis, cardiac hypertrophy, diabetic cardiomyopathy, and cardiac failure. Based on the multiple targets downstream of miRNAs and the complexity of these diseases, a great deal of controversy remains regarding the miRNA-mediated regulation of mitochondria. Therefore, the continued vigorous exploration of miRNAs that regulate mitochondrial function remains necessary to elucidate downstream targets, especially those involved in mitophagy and mitochondrial dynamics. This research will improve our understanding of the biological functions of miRNAs and clarify the crosstalk that occurs between miRNAs and mitochondria. Understanding these networks will provide a theoretical basis for discovering new targets for drugs designed to prevent and treat cardiac disease and provide a new research avenue for clinical cardiac disease.
